# Applying an osteopathic intervention to improve mild to moderate mental health symptoms: a mixed-methods feasibility randomised trial

**DOI:** 10.1186/s12998-024-00556-x

**Published:** 2024-11-06

**Authors:** Josh Hope-Bell, Jerry Draper-Rodi, Darren J. Edwards

**Affiliations:** 1 Department of Public Health, Swansea University, Swansea, UK; 2 Division of Psychological Medicine and Clinical Neurosciences, School of Medicine, Cardiff University, Cardiff, CF10 3AT UK; 3 National Council for Osteopathic Research, Health Science University, London, UK; 4 UCO School of Osteopathy, Health Sciences University, London, UK

**Keywords:** Osteopathic medicine, Mental health, Psychophysiology, Feasibility study, Randomised controlled trial, Heart rate variability, Interoception, Craniosacral therapy, Manual therapy

## Abstract

**Background:**

The increasing prevalence of mental health disorders in the United Kingdom necessitates the exploration of novel treatment modalities. This study aimed to assess the feasibility and acceptability of conducting a randomised controlled trial (RCT) evaluating the efficacy of four osteopathic interventions on psychophysiological and mental health outcomes.

**Methods:**

A mixed-methods feasibility study with an explanatory sequential design was implemented. The quantitative phase involved randomising 42 participants into four intervention groups: (1) high-velocity and articulation techniques (HVAT), (2) soft-tissue massage (STM), (3) craniosacral therapy (CST), and (4) a combination approach. Primary outcome measures encompassed recruitment rate, assessment duration, questionnaire completion, intervention attrition, and adverse events. Secondary outcomes included validated assessments of depression, anxiety, stress, psychological flexibility, heart rate variability (HRV), and interoception, administered pre- and post-intervention. Analysis of variance (ANOVA) was employed to evaluate pre-post intervention changes. The qualitative phase comprised semi-structured interviews analysed using thematic analysis.

**Results:**

The study achieved a recruitment rate of 21 eligible participants per month, with 54.8% of respondents meeting eligibility criteria. All 33 participants who completed the study underwent interventions and assessments within the allocated one-hour timeframe, with full questionnaire completion. The attrition rate was 21%. No adverse events were reported. Qualitative analysis revealed positive participant experiences, with themes highlighting good practitioner communication, intervention accessibility, and increased bodily awareness. Some participants found the questionnaire battery burdensome. Exploratory quantitative analyses showed variations in effects across interventions for heart rate variability, interoceptive accuracy, and mental health measures, but these results should be interpreted cautiously due to the small sample size.

**Conclusions:**

This study provides evidence supporting the feasibility and acceptability of a larger-scale RCT investigating osteopathic interventions for individuals presenting with mild psychological symptoms. The preliminary findings suggest potential efficacy in improving mental health outcomes, warranting further investigation.

*Trial registration* NCT05674071, registered 06/01/2023.

**Supplementary Information:**

The online version contains supplementary material available at 10.1186/s12998-024-00556-x.

## Introduction

Mental health problems such as anxiety and depression are increasingly prevalent [1], and are commonly treated through psychotherapeutic means such as cognitive behavioural therapy (CBT), acceptance and commitment therapy (ACT), as well as pharmacological solutions including antidepressants. While these approaches have demonstrated effectiveness [2, 3], they face significant challenges. High demand for traditional care can lead to difficulties in accessing psychological treatment. Pharmacological treatments can result in unpleasant side effects including dizziness, emotional blunting, and in rare cases, suicidal ideation [4–6]. Talking therapies like CBT and ACT may encounter barriers due to stigma and patient apathy [1, 7]. Given these limitations, there is a pressing need to explore innovative approaches that could provide additional support for mental health services [8]. Such approaches should aim to minimize side effects, reduce stigma, and offer accessible alternatives to traditional treatments.

Recently, it has been suggested that osteopathic interventions could be one such approach to support services as the interventions minimise the impacts of apathy found in talking therapy and offer reduced side effects compared to pharmacological treatments [9, 10]. It is estimated that there are nearly 200,000 clinicians delivering osteopathic care globally, and it is a regulated practice in the USA and in many countries in Europe, including in the UK [11]. An osteopathic approach is patient-centred and focused on the patient’s health rather than disease centred. The practice is evidence informed and scientific rigour forms an important part of treating patients and managing cases [12]. Osteopaths use manual contact to identify and evaluate movement in all structural and functional aspects of the patient, identifying alterations of function and movement that impede health and addressing these [13]. Osteopaths use a variety of techniques to manipulate joints, muscle and tissue. All of the techniques used have an effect on the interplay between the nervous and musculoskeletal systems [14, 15]. Specific techniques include myofascial release, lymphatic drainage, high-velocity, low amplitude (HVLA), articulatory techniques and muscle energy techniques.

Osteopathic techniques may affect the autonomic nervous system (ANS), potentially influencing psychophysiological factors such as heart rate variability (HRV) and interoception [14]. HRV is defined as the variation between consecutive heartbeats [16] and reduced HRV has been associated with depression and anxiety [17–19]. Interoception is the perception of sensations from inside the body such as heartbeat and respiration and ANS activity related to emotion [20]. In terms of anatomy, the interoceptive system consists of afferent neurons which carry sensory information into the spinal laminae I and II. The signals then reach the insular cortex of the brain where they form a pre-stage for emotional awareness. Evidence suggests that impaired interoceptive processing is associated with poorer mental health outcomes [21, 22]. Osteopathy could improve interoception by stimulating Aδ fibres that form part of the interoceptive system’s anatomy [23]. In addition, see Pinna and Edwards for an in-depth review and explanation of interoception and HRV, as well as the association between these factors and mental health [24]. If osteopathic interventions can target these psychophysiological factors, they could in turn have positive impacts on mental health. Experimental studies have demonstrated that osteopathic interventions such as high-velocity thrust, soft-tissue massage and craniosacral therapy can improve interoception and increase HRV [25–29]. The evidence for osteopathic techniques improving psychological symptoms through psychophysiological factors is still limited and the proposed mechanisms are therefore hypothetical, with existing literature being considered purely contextual to this study. However, osteopathy could be a viable approach for supporting mental health services and improving patient’s care and this claim requires further evaluation through a randomised controlled trial (RCT).

### Objectives

This study followed the Medical Research Council (MRC) guidelines for developing and evaluating complex interventions [30]. The main aim was to assess the feasibility of an RCT and the acceptability of an osteopathy intervention for mental health symptoms. A secondary, exploratory aim was to evaluate whether there were any improvements from pre- to post-intervention on standardised psychological questionnaires and psychophysiological measures through exploring effect sizes.

## Methods

This study was a parallel group randomised feasibility study using an explanatory sequential mixed methods approach. In this approach the quantitative aspect formed the first part of the study, followed by the qualitative aspect to help provide further explanation and depth [31]. The MRC states that a purely quantitative approach is rarely sufficient to adequately evaluate interventions [30]. At the feasibility stage, it is crucial to gather detailed insights into participants' experiences with the intervention and assess its acceptability, as this information can guide refinements for future larger-scale trials.

Ethical approval was obtained from the Swansea University Psychology Ethics Committee and the trial has been registered on ClinicalTrials.gov (Identifier: NCT05674071), and the protocol published [32]. The study here has been reported in line with the guidance from the Consolidated Standards of Reporting Trials (CONSORT) extension for randomised pilot and feasibility trials [33]. No important changes were made to the methods after commencement of the trial.

### Design

The study used a parallel, randomised design for the quantitative aspect and semi-structured interviews for the qualitative aspect to assess participant experiences and intervention acceptability.

### Participants

Eligibility criteria included adults with mild to moderate mental health symptoms. Participants with severe mental health scores were excluded to focus specifically on mild to moderate symptoms, and individuals with musculoskeletal pain were excluded to limit potential confounding as a result of alleviated pain symptoms (see Table 1).

**Table 1:** Eligibility criteria for participants recruited into the study

*Inclusion criteria*
1. Age: 18 + years old
2. Mild to moderate score on DASS-21 subscales: Depression = 10–20, Anxiety = 8–14 and Stress = 15–25
3. Ability to speak, read and write English
*Exclusion criteria*
1. Experiencing acute or chronic pain issues at the time of recruitment
2. More severe mental health symptoms as measured by DASS-21 in screening

The study took place at a single site at Swansea University in South Wales, UK. Participants were recruited from both the student population at the university and the general public. The study was advertised through posters around the university and on community noticeboards, social media and student emails. Participants could then follow a QR code to read the information. They could then consent to taking part and answer screening questions for eligibility about their mental health and pain. They were asked if they had experienced any musculoskeletal pain in the last 4 weeks. The osteopath also assessed their pain when they arrived for the intervention.

### Interventions

Participants received one 30-min session of one of four interventions based on osteopathic techniques: (1) high-velocity and articulatory techniques (HVAT), (2) soft-tissue massage, (3) craniosacral techniques, and (4) a combination of all three techniques. The HVAT intervention was applied to the thoracic and rib joints (as is the typical osteopathic approach), followed by hip articulation in extension. The rationale being that these techniques have previously demonstrated autonomic effects [26, 27]. The soft-tissue massage was full-body in prone and supine positions. The craniosacral intervention comprised myofascial release and compression of the fourth ventricle (CV4). Craniosacral techniques are hypothesized to physiologically influence cranial structures which surround the ANS and are important to its maintenance [34]. The combination intervention included HVAT to the thoracic spine, soft-tissue massage of the lower and upper back in prone and CV4. The interventions were delivered by two male practitioners, one with 17 years of practice experience and one with 2 years of practice experience. Both practitioners delivered the four interventions equally. The interventions were all delivered in the same university osteopathy clinic room. The room was neutral to minimise relaxation effects beyond the techniques being delivered. The practitioner communicated with participants during treatment but did not provide mental health advice. A more detailed description of the intervention protocols can be found in the published study protocol [32].

### Outcomes

#### Feasibility

Recruitment feasibility was assessed by response rate to advertisements and eligibility rate after screening, with targets of 100 respondents and 50% eligibility. Measurement feasibility was evaluated by completion time, missing data, retention rate (with ≥ 80% considered acceptable [35]), and equipment setup time for physiological measures.

#### Acceptability

The acceptability of the study has been largely informed by the qualitative interviews following the intervention, the attrition rate and whether any adverse events were reported during or in the subsequent days after the intervention.

### Qualitative data collection

One-to-one interviews were conducted via Microsoft Teams to explore intervention acceptability and impacts. Automated transcription was used and manually verified. Participants were reminded of their rights, including withdrawal and confidentiality. Interviews were conducted post-intervention to avoid influencing quantitative measures. Participants were offered to review the transcript and the opportunity to ask questions before concluding the interview.

### Psychological outcomes

The following measures were collected at pre- and post-intervention:

1. Depression, Anxiety and Stress Scale (DASS-21) which measures three subscales, depression, anxiety and stress [36].
2. International Positive and Negative Affect Schedule- Short-Form (PANAS-SF) which measures positive and negative emotion [37].
3. Acceptance and Action Questionnaire-II (AAQ-II) which measures experiential avoidance, and gives an indication of psychological flexibility [38].
4. Self as Context Scale (SACS) which measures a mindfulness component of psychological flexibility [39].
5. Multidimensional Assessment of Interoceptive Awareness Version 2 (MAIA-2) which measures self-reported bodily or interoceptive awareness [40].

A more detailed description of the measures and their specific psychometric properties can be found in the study protocol [32].

### Physiological outcomes

#### Heart rate variability (HRV)

HRV was measured using a medical-grade Holter electrocardiogram (ECG) monitor. Measurements were taken at two timepoints, immediately pre- and post-intervention. Measurement was conducted in the supine position for 5 min and participants were advised to avoid consuming caffeine, alcohol or nicotine on the day of measurement. A time-domain signal measure was calculated using root mean square of successive interval differences (RMSSD). Frequency-domain measurements were also calculated by using low frequency to high frequency ratio (LF/HF). These HRV measurements were calculated by the software, Kubios (version 3.5) [41]. Estimates of normative values vary but a large-scale review suggested that the range for RMSSD is 19–75 and for LF/HF is 1.1–11.6 [42].

#### Interoceptive accuracy (IAc)

Participants performed a heartbeat counting task as measure of IAc. This was conducted according to the Mental Tracking Method [43] using intervals of 30, 35, 40, and 45 s, separated by 30 s resting periods. This method is widely used in studies where IAc is an outcome measure and has previously been reported as valid and reliable [44, 45]. During each trial R–R intervals are recorded, and participants are asked to silently count their heartbeats without the use of an exteroceptive aid (such as taking one’s pulse). At the end of each period participants verbally report the number of counted heartbeats. The participants are not informed about the length of the counting phases nor about the quality of their performance. The heartbeat counting task was completed pre- and post-intervention. As a guideline, studies have previously considered scores of ≥ 0.70 to be high accuracy and below to be low accuracy [46].

#### Blood pressure (BP)

BP was measured before and after the intervention. This was conducted and interpreted in line with the National Institute for Health and Care Excellence recommendations [47], i.e. with high blood-pressure (HBP) determined by measurements ≥ 140/90 mm Hg. This was done so that the practitioner could assess any undiagnosed HBP, as some osteopathic techniques can be unsafe for individuals with HBP [48].

### Sample size

For the feasibility trial, we aimed to recruit 32 participants. This number of participants is generally deemed sufficient for feasibility studies [49] and would represent approximately 10% of the sample size required in a full trial [50]. We aimed to interview 8–12 participants as part of the qualitative element as suggested by Braun and Clarke for studies of this size [51].

### Randomisation

Participants were randomly assigned to one of the four conditions using a computerised random number generator. Permuted block randomisation was used to reduce imbalances in sample size between conditions. Allocation concealment was ensured using sequentially numbered, sealed opaque envelopes containing the group assignment. Randomisation was implemented by DJE who was not directly involved in data collection or the interventions.

### Blinding

The outcome assessor was blinded to participant’s intervention allocation during data collection and also to intervention allocation while conducting analysis. Further details are provided in the protocol [32].

### Analytical methods

The quantitative aspect had an exploratory aim to observe any exploratory effects of the interventions on outcome measures. After Shapiro–Wilk tests were completed ensuring the data followed a normal distribution (see Supplementary Material 1), ANOVA was used to compare participant’s scores from pre- to post-intervention. The effect sizes are presented as Hedge’s *g,* as this is recommended for smaller sample sizes (*N* < 20) to reduce the risk of overestimating effects [52]. These effect sizes were then entered into G*Power [53] to establish what sample size would be required for a sufficiently powered trial. The full statistical analysis strategy for the quantitative aspect can be found in the study protocol [32].

For the qualitative aspect, thematic analysis (TA) was used to analyse the transcribed interviews [54]. TA is a theoretically and epistemologically flexible method of analysis that seeks to establish patterns in a qualitative dataset. The analysis was therefore conducted following the procedure that is set out by Braun & Clarke [51], which is described in more detail in the study protocol [32].

## Results

**Fig. 1:**
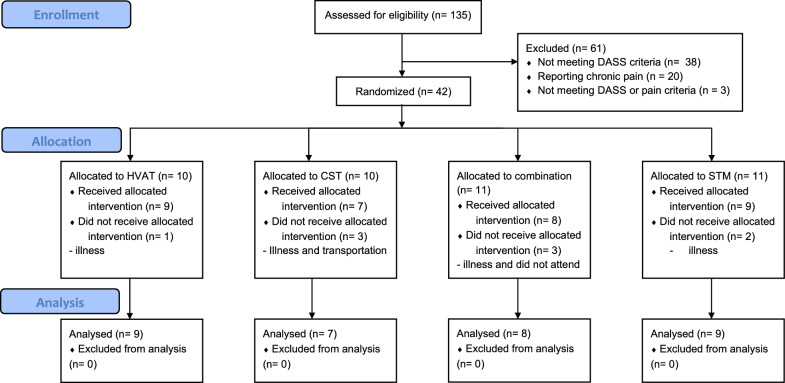
CONSORT flow diagram of participants through the study

Recruitment occurred in November 2022 and February 2023. Of 135 respondents, 74 met the eligibility criteria while 61 were ineligible (38 not meeting DASS criteria, 20 reporting chronic pain, 3 not meeting either criteria). The eligible participants were then contacted to arrange timeslots to attend the intervention on a first come first served basis. 42 participants were randomised to one of the four conditions with 33 participants completing the quantitative stage: HVAT n = 9, STM n = 9, CST n = 7, and combination n = 8 (See Fig. 1).

8 participants completed post-intervention qualitative interviews. A further 4 participants did not take part due to loss of contact and not finding a suitable time to schedule an interview.

Table 2 displays the baseline characteristics of participants who took part in the interventions.

**Table 2:** Demographic characteristics of participants at baseline

	N	%
Baseline characteristic		
Gender		
Male	4	12.1
Female	29	87.9
Ethnicity		
White	26	78.8
Black	2	6.1
Asian	2	6.1
Multiple ethnicities	3	9.1

	*M* (*SD*)	Range
**Age**	24.79 (6.41)	19–45

*M* = mean, *SD* = standard deviation

### Feasibility

The feasibility was determined by the recruitment process and the time taken for data collection. Of the people who responded to advertisements, 42 (54.8%) were eligible providing a recruitment rate of 21 eligible participants per month. Of the 42 randomised participants, 9 people did not attend (for illness and travel issues), providing an attrition rate of 21%. From the qualitative data analysis, data collection procedures were considered generally feasible, though some participants found the number of questions burdensome.

### Acceptability

The qualitative results (discussed in more detail below) suggested that the interventions were acceptable and that participants had a positive experience. There were no recorded adverse events during and after the interventions.

### Qualitative themes

Eight participants took part in these interviews at one-week post-intervention.

The first theme was around practical elements of the interventions, which participants perceived positively. This included good communication from the practitioner, as captured by this quote: “He was nice and friendly. So I think that was quite a bonus because I felt more relaxed.” This points to the acceptability of the intervention and could be an important factor in maintaining retention in a larger trial. Participants also appreciated that the interventions were accessible as characterized by this quote: “So I just thought, well, it's free, so let's do it.” This particular quote suggests that costly treatment may be a barrier to people accessing treatments and so keeping the intervention financially accessible should be considered in a future study.

The second theme encompassed participants' positive perceptions regarding the intervention's impact. Participants seemed to experience a greater sense of relaxation as this quote highlights: “*…because I just found the session really relaxing anyway*”. The environment was not set up to induce extra relaxation, which suggests the osteopathic techniques themselves induced this feeling. Additionally participants reported having a greater awareness of their bodies as this quote suggests: “*And I realised that now I am actually more aware than I had been initially.”* This would suggest that qualitatively, the interventions did have some impact on participant’s interoceptive or mindful awareness levels.

The final theme examined aspects of the research element which participants felt could be improved. This included the questionnaires being burdensome as explained by this quote: “*…But I think I struggle sometimes with when there's a lot of questions that are kind of similar.”* Whilst the measures could be completed in the allotted time, the battery of measures could be reassessed and made more efficient in a larger trial. Participants also expressed some uncertainty around the heartbeat counting task (used as a measure of interoception): “*…but I guess I was surprised by having to count my own heartbeat, mainly because I didn't know how to.”* This points to an aspect of the larger trial that would require more explanation and assurance for participants.

The main themes generated from the analysis have been summarised included in Table 3. Whilst more quotations formed the themes, only the most relevant ones have been included here.

**Table 3:** Main themes, subthemes and representative quotes from qualitative analysis

Subtheme	Description	Quotation
Theme 1: Practical intervention positives		
Patient-practitioner communication	Participants reported feeling that the intervention procedures were explained well but also that the practitioner’s interpersonal style helped put them at ease for the session. This does introduce some potential therapist effects into the interpretation of results. But in similar future research studies that may employ multiple intervention sessions, this could be an important factor in retaining participants	“*He was nice and friendly. So I think that was quite a bonus because I felt more relaxed.”* - Participant 1 “*Yeah… I think he explained it nicely… And I knew what I was expecting.”* Participant 3
Accessibility of the intervention	Participants appreciated that the session of osteopathy was accessible and cited this as a reason for signing up. One participant mentioned that after sharing their experience of the study with others, they responded positively to it being free of charge. Keeping the intervention as accessible as possible could therefore be a key aspect of ensuring sufficient recruitment in future, larger trials of this nature	“*So I just thought, well, it's free, so let's do it.”* Participant 2 “*People I spoke to after said that they would have really valued a free osteopathy session.”* Participant 1
Theme 2: Impact of intervention		
Relaxation from stress	A majority of the participants reported feeling that they were very relaxed after the osteopathy session. It seemed that a number of individuals had signed up due to feeling stressed and so they found this session of osteopathy brought them some relief	“*Yeah, because I do really suffer with stress… I'm interested in finding like techniques and helping with that, so when I saw that I thought I'd give it a go.”* Participant 4 “*…because I just found the session really relaxing anyway*” Participant 1
Greater awareness of body	Participants reported feeling a greater awareness of their body when asked about any changes since the intervention. One participant discussed how the practitioner had informed them that stress can manifest in the back region of the body and that this information stayed with the individual following the session	“*And I realised that now I am actually more aware than I had been initially.”* Participant 1 “*…saying about the top of my back being linked to stress and I know that maybe it made me think about that… When I do feel it I'm like oh, that's because I'm stressed. So I think it's good knowing those sort of links and recognising them in yourself.”* Participant 4
Theme 3: Issues with research element		
Burden of questionnaires	Most participants reported finding the battery of standardised questionnaires too burdensome. There did not seem to be any issues with understanding the content or questions being asked, only the burden of having to respond to the number of questions	“*…But I think I struggle sometimes with when there's a lot of questions that are kind of similar.”* Participant 5 “*I do find them a little bit long…But yeah, I would say a little bit long winded*” Participant 1
Heartbeat counting task being novel	A theme developed whereby participants seemed to pick up on the novelty of the heartbeat counting task. Whereas the ECG and blood pressure were measures that were known to them, they had likely not encountered the task or the notion of interoception before. One participant also reported worrying about being “correct” during the task. It may be that in future studies, further explanation to the participant is needed before the heartbeat counting task to ease any such concerns	“*…but I guess I was surprised by having to count my own heartbeat, mainly because I didn't know how to.”* Participant 3 “*…well I felt very self-conscious about the uh, where I had to count my own heartbeat… I was worried while I was counting. I was like, oh, I'm gonna seem stupid when I give my answer, which obviously like doesn't matter. But then it was affecting my ability to count.”* Participant 6

### Quantitative results

This study is statistically underpowered and so the quantitative results presented are purely speculative in nature. The effect sizes for the psychological and physiological outcome measures from pre-post intervention have therefore been included in Supplementary Material 2. The statistics provide exploratory indications of what within-group changes have occurred for each intervention on the various outcome measures.

### Power analysis

The obtained effect sizes allowed us to conduct G*Power analyses [53], which indicate the sample sizes required to detect meaningful statistical differences for a future RCT. The G*Power (v. 3.1.9.6) indicated that when assuming a between factor (four group), repeated measures (two timepoints) design with an alpha probability of 0.05 and acceptable power of 0.8 [55], then 400 participants are required to detect a meaningful statistical difference for the effect size found in depression, whilst for the other effect sizes, 384 are required for stress, 276 for anxiety and 84 for HRV.

## Discussion

This mixed methods feasibility study found that conducting an RCT of osteopathic interventions for mild-to-moderate mental health symptoms was feasible in terms of recruitment and data collection, and the treatments were acceptable to participants. Qualitative themes highlighted participants' positive perceptions of the practical aspects and impact of the interventions, providing context for the quantitative improvements. However, participants also identified some challenges with the research process, such as the length of questionnaires and novelty of the interoception task. Exploratory analyses also showed effects on psychological and physiological outcomes in certain experimental conditions. However, given the small sample sizes these results should be approached cautiously.

The recruitment figures for the study were encouraging and met the predetermined criteria for feasibility. It suggests an interest in osteopathic approaches and that a larger trial would be possible. Data collection procedures were feasible although it was found that reducing the questionnaire burden would be beneficial. The interventions were also acceptable to participants as reflected in the qualitative analysis and no adverse events were raised. These main outcomes of the study support the implementation of a larger trial in future.

In the qualitative stage, participants reported feeling more relaxed and more aware of their body after the intervention. Relaxation is considered an effective mechanism in reducing depression and anxiety symptoms [56] and may therefore be useful to implement more in pragmatic osteopathic interventions for mental health. Additionally, somatic relaxation has previously been found to reduce cortisol, a physiological marker of stress [57]. This therefore may be an additional physiological measure to consider including in a larger trial. The qualitative results produced additional findings that will be useful in developing future interventions and studies. Participants appreciated that the intervention was accessible. Accessibility to an intervention is an important aspect of its feasibility and acceptability [58] and whilst free treatment may not be possible, the intervention cost should be carefully considered in a larger trial. The participants reported that the communication with the practitioner had been a positive of the intervention. This may lend support to combining osteopathic treatment with talking therapies. Abbey et al. have begun investigating this through the OsteoMAP study [59], which combined ACT with OMT for chronic pain patients. However, such an integrated program could be examined further with pain-free participants experiencing mild mental health symptoms. If the future trial is exploratory in nature, the qualitative results should be used to inform which factors should be tested and which should be included in the placebo intervention to provide a robust mechanistic understanding, or lack thereof, from the results [60].

For the secondary outcomes, combining approaches induced the most significant autonomic relaxation by increasing HRV (RMSSD). This suggests that a variety of techniques focussing on different areas of the body had the most benefit for improving HRV. This reflects common practice in osteopathy in that a combination of techniques are often used in a treatment session [61]. Whilst these findings are exploratory and should be interpreted with caution, they support previous studies demonstrating that osteopathic techniques can have a positive effect on HRV [26, 62]. Self-reporting mental health measures and psychophysiological measures were not fully associated in this study. HVAT produced the largest effects in improvement for self-reported depression, stress and anxiety, and also led to smaller effects on psychophysiology than the other conditions. One explanation for this group reporting larger reductions in symptoms may be that these HVLA techniques were more in line with participants expectations of osteopathic treatment and this intervention therefore felt more salient. The addition of a sham control would aid in further investigation of any effects on psychological and physiological factors. Again these exploratory findings should be approached cautiously and the relationship between self-reported mental health measures and psychophysiological measures is complex and multifaceted [18].

### Strengths and limitations

Our study had some key strengths. Firstly, it was a novel research design in that it examined the utility of several different manual techniques for improving mental health. Previous studies have tended to focus on combined approaches, but the evidence here helps to dissect which techniques can be most usefully applied to reducing mild psychological symptoms. The study also employed rigorous methods including blinding of the outcome assessor and statistician, as well as choosing the intervention techniques based on previous evidence.

There are also some limitations to consider. Firstly, the outcome measures have only been collected over a very brief period. As the primary focus of this study was the feasibility and acceptability of the intervention, longer-term follow-up data was not collected. It is therefore less clear whether the positive effects observed in this study would be sustained over a longer period. An additional limitation stems from the intervention being free of charge to participants. This does not necessarily reflect osteopathic treatment in practice as patients would usually receive care privately and at a cost to themselves. It is therefore less clear how participants would rate the intervention’s acceptability or whether as many participants would sign up if the treatment came at a cost. Likewise, the relaxation effects described in the qualitative analysis should be considered as a potential contextual factor [63]. Also, it would be beneficial to have both male *and* female practitioners delivering the interventions, to be more accommodating to participant’s preferences [64]. These contextual factors should be taken into account when considering the conclusions of this study. Lastly, although some improvements on mental health measures were observed, these may have only been symptom based and therefore temporary. In summary, the study had sound internal validity but had limited external validity.

To improve a larger trial based on this feasibility study, the following strategies should be considered. Targeted marketing through local mental health organisations and social media could be used to facilitate recruitment, and flexible scheduling and clear information about potential benefits could be offered to increase participant interest. The outcome measures would need to be critically evaluated and streamlined, and shorter, validated questionnaires should be explored. The explanation of the interoceptive accuracy task should be improved. The use of a multi-pronged communication approach, with regular check-ins by email and text could help participant retention. Digital platforms that allow participants to complete questionnaires at their own pace within a set timeframe should be explored to assist participants. Long-term follow-up should be considered, such as incorporating 3- and 6-month post-intervention assessments to assess lasting impact. Lastly, the addition of a sham control would help account for contextual factors and provide a meaningful comparison.

## Conclusion

In conclusion, this feasibility trial demonstrates that conducting an RCT of osteopathic interventions for individuals with mild-to-moderate mental health symptoms is both feasible and acceptable. The combination of craniosacral, soft-tissue, and HVAT techniques showed the most promising effects on psychophysiological and psychological outcomes, warranting further investigation in larger, longer-term trials. The integration of qualitative findings provided valuable insights into participants' experiences and perceptions, which can inform the design and refinement of future studies. While this study had some limitations, such as a brief follow-up period and the provision of free treatments, the overall results support the potential of osteopathic interventions as a complementary approach to managing mild-to-moderate mental health symptoms. Further research is needed to establish the efficacy and effectiveness of these techniques in real-world clinical settings.

## Supplementary Information

Additional file 1.

Additional file 2.

## Data Availability

The datasets analysed during the current study are available on the Open Science Framework: https://osf.io/qjw6y/?view_only=d72c72e186694fccabbfa75682c40598.
