# Supplementary material for: Applying an osteopathic intervention to improve mild to moderate mental health symptoms: a mixed-methods feasibility randomised trial
Source: Chiropr Man Therap. 2024 Nov 6;32:32. doi: 10.1186/s12998-024-00556-x (PMC11542205; doi:10.1186/s12998-024-00556-x)
Supplement: Supplementary file 1 — Additional file 1. [file 12998_2024_556_MOESM1_ESM.docx]

**Supplementary Material 1.** Tests of normality for psychological and physiological outcomes

|  | Condition | Shapiro-Wilk | Sig. |
| --- | --- | --- | --- |
| Pre_Depression | CST | .806 | .057 |
|  | Soft Tissue | .917 | .367 |
|  | Combo | .945 | .665 |
|  | HVT | .904 | .276 |
| Post_Depression | CST | .923 | .493 |
|  | Soft Tissue | .941 | .596 |
|  | Combo | .849 | .093 |
|  | HVT | .917 | .365 |
| Pre_Anxiety | CST | .870 | .184 |
|  | Soft Tissue | .890 | .199 |
|  | Combo | .878 | .181 |
|  | HVT | .916 | .357 |
| Post_Anxiety | CST | .950 | .727 |
|  | Soft Tissue | .908 | .303 |
|  | Combo | .966 | .863 |
|  | HVT | .887 | .186 |
| Pre_Stress | CST | .951 | .737 |
|  | Soft Tissue | .868 | .116 |
|  | Combo | .934 | .554 |
|  | HVT | .814 | .060 |
| Post_Stress | CST | .967 | .876 |
|  | Soft Tissue | .874 | .134 |
|  | Combo | .971 | .905 |
|  | HVT | .940 | .579 |

**Table 1.** Psychological outcomes

**Table 2.** Physiological outcomes

|  |  | Statistic | Sig. |
| --- | --- | --- | --- |
| Pre_RMSSD | CST | .960 | .816 |
|  | Soft Tissue | .871 | .127 |
|  | Combo | .835 | .067 |
|  | HVT | .713 | .059 |
| Post_RMSSD | CST | .940 | .638 |
|  | Soft Tissue | .885 | .175 |
|  | Combo | .835 | .067 |
|  | HVT | .707 | .053 |
| Pre_IAC | CST | .970 | .898 |
|  | Soft Tissue | .960 | .798 |
|  | Combo | .903 | .309 |
|  | HVT | .902 | .303 |
| Post_IAC | CST | .862 | .158 |
|  | Soft Tissue | .981 | .967 |
|  | Combo | .931 | .526 |
|  | HVT | .921 | .436 |
