# Supplementary material for: Applying an osteopathic intervention to improve mild to moderate mental health symptoms: a mixed-methods feasibility randomised trial
Source: Chiropr Man Therap. 2024 Nov 6;32:32. doi: 10.1186/s12998-024-00556-x (PMC11542205; doi:10.1186/s12998-024-00556-x)
Supplement: Supplementary file 2 — Additional file 2. [file 12998_2024_556_MOESM2_ESM.docx]

**Supplementary Material 2.** Effect sizes for physiological and psychological outcomes

**HRV**

HRV data was unavailable for one participant in the HVAT condition, as there were significant artefacts when recording at both pre- and post-intervention.

The means and effect sizes for HRV (RMSSD) are summarised below in Table 1. HRV showed the largest pre- / post-intervention effects in the combination group (*g* = 0.93, 95% CI [0.13, 1.69]) and the craniosacral group (*g* = 0.91, 95% CI [0.68, 1.70]). A moderate to large effect size was observed in the soft-tissue group (*g =* 0.76, 95% CI [0.50, 1.44]), and small effect size in the HVAT group (*g* = 0.16, 95% CI [-0.46, 0.78]). Frequency-domain HRV was measured using low-frequency to high-frequency ratio (LF/HF). A moderate effect size was observed in the craniosacral group (*g* = 0.54, 95% CI [-0.19, 1.23]), small effect sizes in the combination group (*g =* 0.44, 95% [-0.23, 1.08]), the HVAT group (*g* = 0.33, 95% CI [-0.32, 0.96]) and a very small effect in the soft-tissue group (*g* = 0.18, 95% CI [-0.42, 0.77]). In all groups, LF/HF ratio decreased suggesting greater psychophysiological relaxation. RMSSD is considered the most reliable measure of HRV (51) and so the increases on this measure are of particular note. The means and effect sizes for HRV (RMSSD) are summarised below in Table 1.

**Table 1.** Mean scores and effect sizes for HRV (RMSSD)

| **Condition** | **Pre HRV** | **SD** | **Post HRV** | **SD** | **Effect size (Hedge's *g*)** | **95% CI** |
| --- | --- | --- | --- | --- | --- | --- |
| Craniosacral | 42.46 | 17.41 | 54.09 | 13.88 | 0.91 | [0.68, 1.70] |
| Soft-tissue | 47.90 | 30.16 | 66.71 | 38.15 | 0.76 | [0.50, 1.44] |
| Combination | 43.48 | 23.16 | 48.03 | 23.42 | 0.93 | [0.13, 1.69] |
| HVAT | 45.94 | 35.76 | 48.02 | 34.37 | 0.16 | [-0.46, 0.78] |

*Note*. SD = standard deviation, CI = confidence interval. HVAT n = 8, STM n = 9, CST n = 7, and combination n = 8.

**Interoceptive accuracy (IAc)**

Decreases in IAc were observed with a moderate to large effect size in the soft-tissue group (*g* = 0.79, 95% CI [0.07, 1.47) and a very small effect size in the craniosacral group (*g =* 0.18, 95% CI [-0.48, 0.82]). Very small increases in IAc were observed in the HVAT group (*g =* 0.02, 95% CI [-0.61, 0.57]) and the combination group (*g =* 0.01, 95% CI [-0.63, 0.60]).

**Mental health**

The results for psychological outcomes are summarised in Table 2 below. For mental health symptoms, the craniosacral group showed a small effect size for stress (*g =* 0.28, 95% CI [-0.39, 0.93]) while very small effect sizes were found for reductions in depression (*g =* 0.18, 95% CI [-0.48, 0.82]) and anxiety (*g =* 0.03, 95% CI [-0.62, 0.67]). In the soft-tissue condition, small effect sizes were found for reductions in anxiety (*g =* 0.41, 95% CI [-0.22, 1.02], stress (*g =* 0.29, 95% CI [-0.33, 0.88]), and depression (*g =* 0.32, 95% CI [-0.30, 0.92]). The combination group showed small effect sizes for reductions in anxiety (*g =* 0.28, 95% CI [-0.39, 0.93]) and stress (*g =* 0.26, 95% CI [-0.41, 0.90]), and a very small effect size for reduction in depression (*g =* 0.14, 95% CI [-0.51, 0.78]). Lastly, the HVAT group showed a large effect size for reductions in depression (*g =* 0.89, 95% CI [0.14, 1.60]) and anxiety (*g =* 0.78, 95% CI [0.67, 1.47]) and a moderate effect size was found for reduction in stress (*g =* 0.54, 95% CI [-0.12, 1.17]).

**Table 2.** Means and effect sizes for self-reported depression, anxiety and stress scores.

|  | **Condition** | | | |
| --- | --- | --- | --- | --- |
| **Measure** | ***Craniosacral*** | ***Soft-tissue*** | ***Combination*** | ***HVAT*** |
| Depression pre | 8.00 | 10.11 | 9.86 | 11.33 |
| Depression post | 7.29 | 8.22 | 9.00 | 7.78 |
| Effect size (Hedge’s *g*) | 0.18 | 0.32 | 0.14 | 0.89 |
| 95% CI | [-0.48, 0.82] | [-0.30, 0.92] | [-0.51, 0.78] | [0.14, 1.60] |
| Anxiety pre | 7.57 | 7.33 | 9.00 | 9.22 |
| Anxiety post | 7.43 | 5.78 | 8.29 | 5.22 |
| Effect size (Hedge’s *g*) | 0.03 | 0.41 | 0.28 | 0.78 |
| 95% CI | [-0.62, 0.67] | [-0.22, 1.02] | [-0.39, 0.93] | [0.67, 1.47] |
| Stress pre | 10.71 | 13.44 | 12.71 | 13.89 |
| Stress post | 9.29 | 11.78 | 11.57 | 10.22 |
| Effect size (Hedge's *g*) | 0.28 | 0.29 | 0.26 | 0.54 |
| 95% CI | [-0.39, 0.93] | [-0.33, 0.88] | [-0.41, 0.90] | [-0.12, 1.17] |

*Note.* Pre = pre-intervention, post = post-intervention. Lower scores indicate lower levels of depression, anxiety or stress. CI = confidence interval. HVAT n = 9, STM n = 9, CST n = 7, and combination n = 8.
